# Validation of a Proprietary Deterioration Index Model and Performance in Hospitalized Adults

**DOI:** 10.1001/jamanetworkopen.2023.24176

**Published:** 2023-07-24

**Authors:** Thomas F. Byrd, Bronwyn Southwell, Adarsh Ravishankar, Travis Tran, Abhinab Kc, Tom Phelan, Genevieve B. Melton-Meaux, Michael G. Usher, Daren Scheppmann, Sean Switzer, Gyorgy Simon, Christopher J. Tignanelli

**Affiliations:** 1 Department of Medicine, University of Minnesota, Minneapolis; 2 Institute for Health Informatics, University of Minnesota, Minneapolis; 3 Department of Anesthesiology, University of Minnesota, Minneapolis; 4 Department of Dermatology, University of Minnesota, Minneapolis; 5 University of Minnesota Medical School, University of Minnesota, Minneapolis; 6 Fairview Health Services, Minneapolis, Minnesota; 7 Department of Surgery, University of Minnesota, Minneapolis; 8 Center for Learning Health System Sciences, University of Minnesota, Minneapolis

## Abstract

**Importance:**

The Deterioration Index (DTI), used by hospitals for predicting patient deterioration, has not been extensively validated externally, raising concerns about performance and equitable predictions.

**Objective:**

To locally validate DTI performance and assess its potential for bias in predicting patient clinical deterioration.

**Design, Setting, and Participants:**

This retrospective prognostic study included 13 737 patients admitted to 8 heterogenous Midwestern US hospitals varying in size and type, including academic, community, urban, and rural hospitals. Patients were 18 years or older and admitted between January 1 and May 31, 2021.

**Exposure:**

DTI predictions made every 15 minutes.

**Main Outcomes and Measures:**

Deterioration, defined as the occurrence of any of the following while hospitalized: mechanical ventilation, intensive care unit transfer, or death. Performance of the DTI was evaluated using area under the receiver operating characteristic curve (AUROC) and area under the precision recall curve (AUPRC). Bias measures were calculated across demographic subgroups.

**Results:**

A total of 5 143 513 DTI predictions were made for 13 737 patients across 14 834 hospitalizations. Among 13 918 encounters, the mean (SD) age of patients was 60.3 (19.2) years; 7636 (54.9%) were female, 11 345 (81.5%) were White, and 12 392 (89.0%) were of other ethnicity than Hispanic or Latino. The prevalence of deterioration was 10.3% (n = 1436). The DTI produced AUROCs of 0.759 (95% CI, 0.756-0.762) at the observation level and 0.685 (95% CI, 0.671-0.700) at the encounter level. Corresponding AUPRCs were 0.039 (95% CI, 0.037-0.040) at the observation level and 0.248 (95% CI, 0.227-0.273) at the encounter level. Bias measures varied across demographic subgroups and were 14.0% worse for patients identifying as American Indian or Alaska Native and 19.0% worse for those who chose not to disclose their ethnicity.

**Conclusions and Relevance:**

In this prognostic study, the DTI had modest ability to predict patient deterioration, with varying degrees of performance at the observation and encounter levels and across different demographic groups. Disparate performance across subgroups suggests the need for more transparency in model training data and reinforces the need to locally validate externally developed prediction models.

## Key Points

**Question:**

How does the Deterioration Index perform in predicting patient clinical deterioration across diverse hospital settings and demographic groups?

**Findings:**

In this prognostic study involving more than 5 million DTI predictions for 13 737 patients, the DTI had acceptable discrimination at the observation level but poor discrimination at the encounter level. Performance also varied by demographic subgroup, with worse performance for patients identifying as American Indian or Alaska Native and those who chose not to disclose their ethnicity.

**Meaning:**

These findings highlight the need to consider patient demographic characteristics and variations in care practices when implementing predictive models such as the DTI.

## Teaser

This prognostic study evaluates and validates the performance and potential for bias of a proprietary deterioration index in predicting clinical deterioration among hospitalized adult patients.

## Introduction

Failure to recognize or act upon clinical deterioration is associated with an estimated 15% of avoidable deaths in the hospital.^1^ While nurses and physicians often do not recognize deterioration, their performance can improve when presented with information from an electronic early warning system.^2,3,4^ However, traditional scoring systems such as the National Early Warning System are neither sensitive nor specific and consequently produce alert fatigue.^3,5^ In response, model developers have turned to machine learning to gain better predictive performance.^6^

The Deterioration Index (DTI; Epic Systems Corporation; hereinafter, Epic) is a proprietary machine learning model that has been adopted across hundreds of hospitals since its release in 2017.^7^ Given widespread use of the DTI to assist in care delivery, it is important to measure its performance across as many health care settings as possible.^8^ Notably, the optimal DTI score at which to trigger clinical interventions remains unclear, and the vendor does not report performance across subgroups of race, ethnicity, age, or sex.^9^ This study aimed to (1) describe the overall and lead time performance of the DTI among a large cohort of patients across 8 heterogenous Midwestern US hospitals, (2) evaluate performance measures at suggested thresholds to support clinical decision-making, and (3) assess bias in predictions among demographic subgroups.

## Methods

This prognostic study was approved by the institutional review board of the University of Minnesota, which waived the requirement for consent because there was no more than minimal risk to participants, no way to practically conduct the research without the waiver, and no adverse effect on the rights and welfare of the participants without the waiver. Only patients who had not chosen to opt out of research during their initial interaction with our health care system, such as a first clinic appointment or emergency department registration, were included in the study. This study followed the Transparent Reporting of a Multivariable Prediction Model for Individual Prognosis or Diagnosis (TRIPOD) reporting guideline for prediction model validation.

### The Deterioration Index

The DTI is a proprietary ordinal logistic regression model that was trained to predict patient clinical deterioration, defined as escalation of care (intensive care unit [ICU] transfer, rapid response team [RRT] activation, or code team activation) within 12 hours or death (defined as discharge time) within 38 hours.^10^ The model outputs a probabilistic score between 0 and 100 every 15 minutes, with a higher score indicating higher risk of deterioration. Detailed demographic information used for the training data set are not available publicly. A list of variables used by the DTI is provided in eMethods in Supplement 1.

### Setting and Study Population

In 2020, our organization silently turned on the DTI model, which ran in the background of our electronic health record (EHR). Consequently, clinicians did not see or act on the scores generated by the DTI. For this study, we included all patients 18 years or older who were hospitalized at MHealth Fairview (the health system of the University of Minnesota) between January 1 and May 31, 2021, and who had at least 1 DTI score recorded. The 8 hospitals in our study were a mix of academic (n = 2) and community (n = 6) hospitals, including 3 rural hospitals (as defined by the Health Resources and Services Administration rural-urban commuting area code criteria).^11^ We excluded predictions made in preoperative, intraoperative, ICU, or labor and delivery locations because surgical procedures were likely to involve planned mechanical intubation, ICU patients had already deteriorated, and labor and delivery patients were excluded from the DTI training data.

### Definition of Outcomes

We defined our primary outcome, deterioration, as mechanical ventilation, ICU transfer, or death (defined as time of discharge).^12,13,14,15,16^ Deterioration events occurring after comfort care orders were excluded from analyses. Although the DTI was trained to also detect RRT or code team activations, our EHR did not discretely capture these events; consequently, they could not be incorporated into our definition of deterioration. Nevertheless, to gain insights into the occurrence of such events in patients experiencing deterioration across our hospitals, we conducted a manual records review of 50 cases (eMethods and eTable 2 in Supplement 1). This approach, although exploratory, allowed us to better understand the underlying factors associated with deterioration, the preventability of deterioration, and the frequency of RRT and code team activations among patients experiencing deterioration.

### Model Validation

We used area under the receiver operating characteristic curve (AUROC) and area under the precision recall curve (AUPRC) to describe the overall performance of the DTI among the patient population. The AUROC is the probability that the model correctly ranks deterioration risk among 2 randomly selected samples from the outcome and nonoutcome groups. The AUPRC is the weighted mean of precisions (ie, positive predictive values [PPVs]) achieved at each threshold, in which weight is the increase in recall (ie, sensitivity) from the previous threshold.

We used both observation- and encounter-level evaluation methods for our primary analysis, consistent with existing evaluation studies of deterioration prediction models^16,17,18,19,20^ (eMethods in Supplement 1). At the observation level, we included all DTI scores across each hospitalization and asked whether the outcome occurred within 12 hours of each prediction. At the encounter level, we included only the highest DTI score from each hospitalization and asked whether the outcome occurred any time thereafter. In all cases, scores derived after deterioration, including scores in the ICU, were excluded. We tested model performance at the observation level across several lead times (3 hours, 6 hours, 12 hours, 24 hours, 38 hours, and 72 hours)^16,17,21,22^ for the composite definition of deterioration and each of its component events. Calibration was visualized by plotting calibration curves with 20 bins each.^23^

### Threshold Analysis

As an ordinal model, the DTI was designed to predict 2 distinct events: care escalation and death. To accommodate these different outcomes, the vendor allows hospitals to set both a medium- and high-risk score threshold. Accordingly, we evaluated DTI performance at both a medium-risk threshold targeting 50% sensitivity and a high-risk threshold targeting 10% PPV. Hospitals may also prefer a single threshold to trigger an intervention,^24^ in which striking a balance between sensitivity and PPV is essential. We used the maximum F1 score (the harmonic mean of sensitivity and PPV) to identify an optimal single score threshold. We report the number needed to evaluate (NNE) at each threshold, calculated as the inverse of PPV. The NNE describes the number of predictions with a score higher than the threshold that must be evaluated to identify 1 case of deterioration.^25^

### Bias Assessment

We evaluated the ability of the DTI to make unbiased predictions by calculating bias measures across subgroups of race, ethnicity, biological sex, and age. Race and ethnicity were self-reported in the EHR. Race and ethnicity data were relevant to the assessment of bias because disparities in deterioration among certain racial and ethnic groups may be perpetuated or worsened by biased algorithmic predictions.^26,27^ Ethnicity was reduced from more than 100 options (eTable 1 in Supplement 1) into 2 categories, Hispanic or Latino (hereinafter, Hispanic) or other ethnicity, with the latter category including all those who did not report Hispanic ethnicity. Age was dichotomized to younger than 60 years or 60 years or older.

To measure potential bias, we calculated 3 bias measures^28^ for each subgroup. Within each subgroup, we defined the protected group as the subset that did not identify as White race, other ethnicity, male sex, or age 60 years or older. First, we calculated AUROC parity, defined as the ratio of the AUROC of the protected group to the AUROC of the reference group. Second, we calculated sensitivity parity (also known as equal opportunity), defined as the ratio of the model’s sensitivity in the protected group to its sensitivity in the reference group. Third, we calculated PPV parity (also known as false discovery rate parity), defined as the ratio of the model’s PPV in the protected group to its PPV in the reference group. We studied these 3 measures because a deterioration model must discriminate well (AUROC parity) while identifying the greatest number of patients experiencing deterioration (sensitivity parity) and avoiding false-positive cases (PPV parity). We measured sensitivity and PPV parity at the threshold that maximized F1 score.

### Statistical Analysis

We abstained from conducting formal statistical tests on model performance measures because our focus was solely on evaluating a single model. We calculated 95% CIs for proportional point estimates using the Wilson score.^29^ Differences in model performance across lead times and subgroups should be considered exploratory. All analyses were performed using Stata software, version 16.1 (StataCorp LLC); Python software, version 3.9 (Python Software Foundation); and R software, version 4.2.3 (R Foundation for Statistical Computing).

## Results

**Figure 1.**
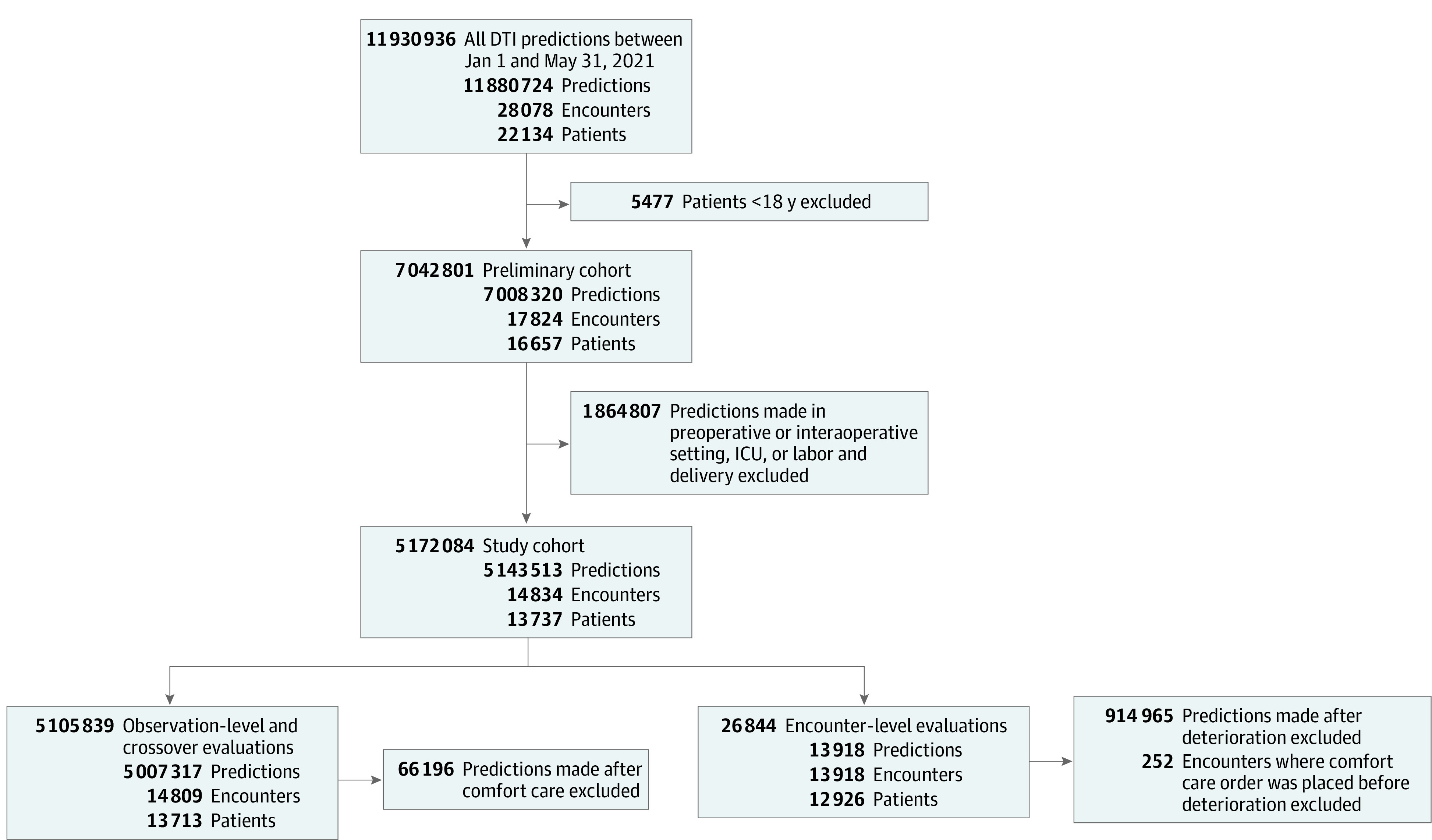
Flowchart of Patient Selection and Inclusion and Exclusion Criteria DTI indicates Deterioration Index; and ICU, intensive care unit.

A total of 13 737 patients from 8 hospitals, representing 14 834 encounters and 5 143 513 DTI predictions, met the inclusion criteria (Figure 1). Once we removed predictions made after deterioration and encounters in which comfort care orders preceded deterioration, the remaining data comprised 13 918 encounters for analysis at the encounter level. Among 13 918 encounters, the mean (SD) age of patients was 60.3 (19.2) years; 7636 (54.9%) were female, and 6282 (45.1%) were male (Table 1). With regard to race, 247 patients (1.8%) were American Indian or Alaska Native, 434 (3.1%) were Asian, 1255 (9.0%) were Black or African American (hereinafter, Black), 11 (0.1%) were Middle Eastern or North African, 22 (0.2%) were Native Hawaiian or other Pacific Islander, 11 345 (81.5%) were White, and 568 (4.1%) chose not to answer; data on race were missing for 36 encounters (0.3%). With regard to ethnicity, 255 (1.8%) were Hispanic, and 12 392 (89.0%) were of other ethnicity (eTable 1 in Supplement 1). Deterioration occurred in 1436 encounters (10.3%), with no deterioration occurring in 12 482 encounters (89.7%). Compared with patients who did not experience deterioration, those with deterioration were more likely to be male (789 patients [54.9%] vs 5493 [44.0%]), have comorbid conditions (eg, fluid and electrolyte disorder: 1126 patients [78.4%] vs 7617 [61.0%]; neurological disorder: 712 patients [49.6% ] vs 4422 [35.4%]), and have a sepsis discharge diagnosis (356 patients [24.8%] vs 1152 [9.2%]). The median (IQR) length of stay for patients who experienced deterioration was 5.3 (2.9-10.8) days vs 3.0 (1.9-4.8) days for patients who did not experience deterioration. The median (IQR) time from presentation to deterioration was 68 (39-109) hours. Patients who had an admission code status of full code experienced deterioration at a rate comparable with those who had do not resuscitate orders (1288 of 12 049 patients [10.7%] vs 153 of 1392 [11.0%]).

**Table 1.** Characteristics of Patients Across Hospitalizations

Characteristic	Hospitalizations, No. (%)		
	Overall (n = 13 918)	No deterioration (n = 12 482)	Deterioration (n = 1436)
Age, mean (SD), y	60.3 (19.2)	60.2 (19.4)	61.2 (17.2)
Sex			
Female	7636 (54.9)	6989 (56.0)	647 (45.1)
Male	6282 (45.1)	5493 (44.0)	789 (54.9)
Race			
American Indian or Alaska Native	247 (1.8)	214 (1.7)	33 (2.3)
Asian	434 (3.1)	391 (3.1)	43 (3.0)
Black or African American	1255 (9.0)	1151 (9.2)	104 (7.2)
Middle Eastern or North African	11 (0.1)	9 (0.1)	2 (0.1)
Native Hawaiian or other Pacific Islander	22 (0.2)	20 (0.2)	2 (0.1)
White	11 345 (81.5)	10 150 (81.3)	1195 (83.2)
Chose not to answer	568 (4.1)	516 (4.1)	52 (3.6)
Missing	36 (0.3)	31 (0.2)	5 (0.3)
Ethnicity			
Hispanic or Latino	255 (1.8)	231 (1.9)	24 (1.7)
Other ethnicity^a^	12 392 (89.0)	11 088 (88.8)	1304 (90.8)
Chose not to answer	1271 (9.1)	1163 (9.3)	108 (7.5)
Sepsis discharge diagnosis	1508 (10.8)	1152 (9.2)	356 (24.8)
Length of stay, median (IQR), d	3.1 (2.0-5.1)	3.0 (1.9-4.8)	5.3 (2.9-10.8)
Code status on admission			
Full	12 049 (86.6)	10 821 (86.7)	1228 (85.5)
DNR^b^	1392 (10.0)	1239 (9.9)	153 (10.7)
Missing	477 (3.4)	422 (3.4)	55 (3.8)
Comorbidities			
Congestive heart failure	3745 (26.9)	3232 (25.9)	513 (35.7)
Pulmonary circulation disorder	2030 (14.6)	1770 (14.2)	260 (18.1)
Peripheral vascular disorder	3935 (28.3)	3443 (27.6)	492 (34.3)
Neurological disorder	5134 (36.9)	4422 (35.4)	712 (49.6)
Chronic pulmonary disease	5071 (36.4)	4470 (35.8)	601 (41.9)
Diabetes, uncomplicated	4119 (29.6)	3608 (28.9)	511 (35.6)
Diabetes, complicated	3647 (26.2)	3173 (25.4)	474 (33.0)
Peptic ulcer disease	1211 (8.7)	1078 (8.6)	133 (9.3)
Solid tumor without metastasis	3328 (23.9)	2953 (23.7)	375 (26.1)
Rheumatoid arthritis or collagen vascular disease	1714 (12.3)	1543 (12.4)	171 (11.9)
Coagulopathy	4028 (28.9)	3464 (27.8)	564 (39.3)
Fluid and electrolyte disorder	8743 (62.8)	7617 (61.0)	1126 (78.4)

Abbreviation: DNR, do not resuscitate.

^a^ Ethnicities included in the *other ethnicity* category are provided in eTable 1 in Supplement 1.

^b^ Includes DNR status, DNR and do not intubate status, and special code (do not perform cardiopulmonary resuscitation but intubation allowed) status.

Through records review, we identified 2 cardiac arrests among the 50 total cases of deterioration. A rapid response was called on 7 patients (14.0%), all of whom eventually experienced deterioration. The underlying reason for deterioration in 2 patients (4.0%) was clearly COVID-19. Bacterial sepsis was clearly the underlying reason for deterioration in 4 patients (8.0%). In all, 16 of 43 deterioration events (37.2%) were deemed unlikely preventable while 23 (53.5%) were deemed clearly not preventable.

### Overall and Lead Time Performance

At the observation level, the DTI had an AUROC of 0.759 (95% CI, 0.756-0.762) and an AUPRC of 0.039 (95% CI, 0.037-0.040), suggesting acceptable discrimination. At the encounter level, the DTI had an AUROC of 0.685 (95% CI, 0.671-0.700) and an AUPRC of 0.248 (95% CI, 0.227-0.273), suggesting poor discrimination. The AUROC and AUPRC plots are shown in eFigure 1 in Supplement 1. Calibration curves (eFigures 2 and 3 in Supplement 1) revealed that the DTI consistently produced disproportionately high risk estimates.

The ability of the DTI to predict the individual components and composite definition of deterioration across increasing lead times, evaluated at the observation level, is shown in Table 2. For mechanical ventilation, the AUROC decreased from 0.848 (95% CI, 0.838-0.859) at 3 hours to 0.677 (95% CI, 0.674-0.680) at 72 hours. For ICU transfer, the AUROC decreased from 0.784 (95% CI, 0.780-0.788) at 3 hours to 0.681 (95% CI, 0.679-0.683) at 72 hours. For death, the AUROC was highest at 24 hours, with a value of 0.877 (95% CI, 0.872-0.882), and remained relatively high across all time horizons, ranging from 0.863 (95% CI, 0.844-0.882) at 3 hours to 0.868 (95% CI, 0.866-0.871) at 72 hours. For the composite definition of deterioration (the first of mechanical ventilation, ICU transfer, or death), the AUROC decreased from 0.782 (95% CI, 0.777-0.786) at 3 hours to 0.707 (95% CI, 0.706-0.709) at 72 hours.

**Table 2.** Performance Across Lead Times by Deterioration Event

Deterioration event	AUROC (95% CI)					
	3 h	6 h	12 h	24 h	38 h	72 h
Mechanical ventilation	0.848 (0.838-0.859)	0.836 (0.828-0.843)	0.783 (0.777-0.789)	0.728 (0.723-0.732)	0.702 (0.698-0.705)	0.677 (0.674-0.680)
ICU transfer	0.784 (0.780-0.788)	0.782 (0.778-0.786)	0.759 (0.756-0.762)	0.719 (0.716-0.722)	0.700 (0.697-0.702)	0.681 (0.679-0.683)
Death	0.863 (0.844-0.882)	0.865 (0.852-0.877)	0.872 (0.864-0.880)	0.877 (0.872-0.882)	0.880 (0.876-0.883)	0.868 (0.866-0.871)
Mechanical ventilation, ICU transfer, or death	0.782 (0.777-0.786)	0.780 (0.776-0.783)	0.759 (0.756-0.762)	0.728 (0.725-0.730)	0.715 (0.713-0.717)	0.707 (0.706-0.709)

Abbreviations: AUROC, area under the receiver operating characteristic curve; ICU, intensive care unit.

### Threshold Analysis

Observation-level performance measures at clinically informed score thresholds are shown in Table 3. We deliberately selected a suggested medium-risk score threshold (37.4) to achieve a sensitivity of 50% based on our consensus that being able to correctly identify approximately one-half of the true-positive cases would provide substantial value in a clinical setting. At this threshold, only 2.1% (95% CI, 2.1%-2.1%) of positive predictions were true-positive cases; a clinician would have to view 48 predictions (NNE, 47.6; 95% CI, 46.6-48.1) to identify 1 true-positive case. At our suggested single score threshold (63.9), which was set at the model’s maximal F1 score, PPV improved to 8.2% (95% CI, 7.9%-8.4%), while sensitivity decreased to 10.8% (95% CI, 10.5%-11.2%). We deliberately selected a suggested high-risk score threshold (68.3) to achieve a PPV of 10%, which was the highest PPV that the DTI was reasonably able to produce. At this threshold, a clinician would have to view 10 predictions (NNE, 10.0; 95% CI, 9.6-10.4) to find 1 true-positive case, while only 7.9% (95% CI, 7.6%-8.3%) of patients with deterioration would be identified.

**Table 3.** Suggested Score Threshold Performance^a^

Performance measure	Suggested medium-risk threshold	Suggested single threshold	Suggested high-risk threshold
Goal	Emphasize sensitivity	Maximize F1 score (balance sensitivity and PPV)	Emphasize PPV
Score threshold	37.4	63.9	68.3
F1 score, % (95% CI)	4.1 (4.0-4.1)	9.3 (9.3-9.3)	8.9 (8.8-8.9)
Sensitivity, % (95% CI)	50.2 (49.6-50.8)	10.8 (10.5-11.2)	7.9 (7.6-8.3)
Specificity, % (95% CI)	84.5 (84.5-84.6)	99.2 (99.2-99.2)	99.5 (99.5-99.5)
PPV, % (95% CI)	2.1 (2.1-2.1)	8.2 (7.9-8.4)	10.0 (9.6-10.4)
NPV, % (95% CI)	99.6 (99.6-99.6)	99.4 (99.4-99.4)	99.4 (99.4-99.4)
NNE	47.6 (46.6-48.1)	12.3 (11.9-12.7)	10.0 (9.6-10.4)

Abbreviations: NNE, number needed to evaluate; NPV, negative predictive value; PPV, positive predictive value.

^a^ Observation-level measures are shown (4 183 737 total predictions; deterioration prevalence of 0.7%).

### Bias Assessment

**Figure 2.**
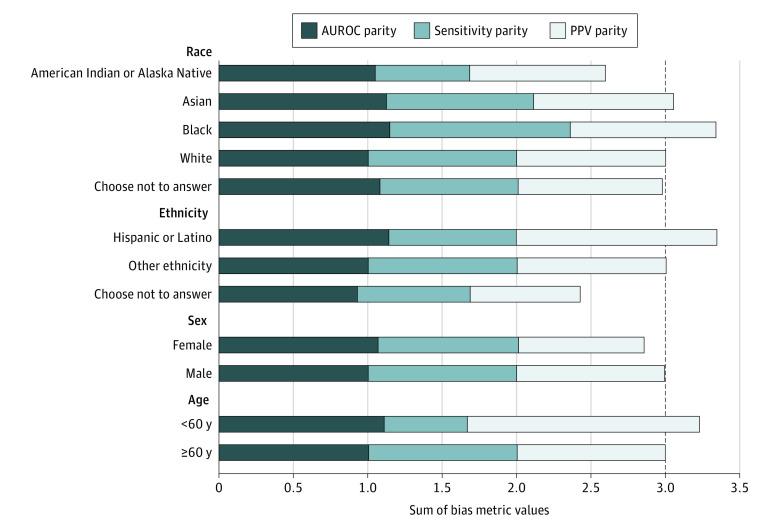
Variation in Model Performance Variation was measured by the sum of 3 bias measures across patient subgroups. Data were measured at the encounter level. Reference group parity values were equal to 1 for each individual bias measure within each subgroup. Reference groups were White patients, patients who identified as other ethnicity, male patients, and patients 60 years or older. The dashed line represents the reference group parity value for the sum of all 3 bias measures. AUROC indicates area under the receiver operating characteristic curve; and PPV, positive predictive value.

Bias measures for protected subgroups are shown in Figure 2. All racial subgroups exhibited PPV parity close to or slightly lower than that of White patients (eg, Black patients: 0.97). Those identifying as Hispanic and those younger than 60 years had higher PPV parity (1.35 and 1.56, respectively). Sensitivity parity was lower in patients who identified as American Indian or Alaska Native (0.64), those younger than 60 years (0.56), and those who chose not to disclose their ethnicity (0.76). Sensitivity parity was higher among Black patients (1.21) compared with White patients. The AUROC parity was higher than 1.00 for all groups except those who chose not to disclose their ethnicity (0.93). Combined, parity was 14.0% worse for American Indian or Alaska Native patients and 19.0% worse for patients who chose not to disclose their ethnicity. Conversely, parity was 11.0% better for Black and Hispanic patients.

## Discussion

The purpose of this prognostic study was to comprehensively evaluate the performance of the DTI among a large cohort of patients across 8 heterogenous midwestern US hospitals. We discovered 3 key findings: (1) the DTI had acceptable discrimination in predicting deterioration at the observation level and poor discrimination at the encounter level, with discrimination improving closer to deterioration; (2) achieving clinically appropriate DTI score thresholds is challenging due to large tradeoffs between sensitivity and PPV; and (3) DTI performance varied across certain demographic subgroups.

Overall DTI performance fluctuated widely by encounter- vs observation-level evaluation methods, as it has in other settings. For example, Epic initially used an encounter-level evaluation method to validate the DTI and reported its mean AUROC as 0.801.^10^ In late 2022, Epic switched to an observation-level evaluation method because it “more accurately represents the experience clinicians have when using the model.”^10^ Epic currently reports a mean AUROC of 0.710 (used with permission),^10^ which lies between our calculated observation- and encounter-level AUROCs. Compared with the largest existing external validation of the DTI,^20^ our observation-level AUROC was similar (0.759 vs 0.768-0.780), while our encounter-level AUROC was substantially lower (0.685 vs 0.821-0.863). We found a general pattern of decreasing performance with increasing lead times for mechanical ventilation and ICU transfer, whereas performance for death remained relatively high and stable across lead times. Overall performance was more reliable in the immediate term (within a few hours) and decreased over longer periods. The variable performance of the DTI across different components of deterioration highlights the necessity of context-specific interpretation.

Our strategic selection of suggested score thresholds revealed large tradeoffs in sensitivity and PPV. At the medium-risk score threshold, we prioritized sensitivity to ensure that a substantial proportion of patients at risk was identified. However, the high NNE at this threshold could lead to resource strain due to a high number of false-positive cases. By optimizing the model’s maximal F1 score, we balanced sensitivity and PPV to create a single decision threshold. A single-threshold approach is best suited for a well-calibrated model; however, our calibration curves revealed that the DTI does not exhibit a linear association between predicted risk and deterioration. Given the ordinal nature of the model, poor calibration may be expected, which supports the use of 2 distinct score thresholds. Accordingly, we chose our high-risk threshold to prioritize PPV at the expense of identifying a substantially smaller proportion of patients at risk.

Bias in predictive models manifests as a systematic favoritism in the model’s predictions, often because of insufficient data collection, selection, or model training.^30^ While bias is suggested if parity measure values approach 0.2 with regard to the reference group, the interpretation of such inequality in parity must be contextually aligned with the specific application and potential implications of the observed disparity.^31^ For example, although our results suggested that the DTI performed worse among American Indian or Alaska Native patients, with an overall parity discrepancy of 14.0%, this performance could be associated with differences in care with respect to our definition of deterioration rather than inherent statistical bias present in the model. Our system might send White patients to the ICU faster and more frequently than American Indian or Alaska Native patients who have the same severity of illness. An external model trained on nonbiased data could accurately predict the same risk of ICU transfer for both groups; however, when comparing their rates for postprediction ICU transfers, the model would appear to overestimate risk for White patients and underestimate risk for American Indian or Alaska Native patients. In contrast, we found that the DTI exhibited performance parity among Black and Hispanic patients, 2 groups traditionally at risk of machine learning bias due to poor representation in training data. Notably, we found the largest parity discrepancy of 19.0% for patients who chose not to disclose their ethnicity, and future research should explore whether this nondisclosure represents a distinct clinical risk profile.

Our records review revealed no instances of preventable deterioration because most patients were known to be critically ill or had been appropriately transferred to the ICU for closer monitoring. Despite the small sample, our findings raise a pertinent clinical question: how often do DTI predictions provide new and actionable insights for the health care team? Higher risk thresholds may overlook patients who could benefit from early intervention. Consequently, future research is needed to formulate standard measures that could answer this question both retrospectively and prospectively during model implementation.

### Limitations

This study has several limitations. We did not stratify our analysis by COVID-19 positivity. Because the DTI was developed before the emergence of COVID-19, researchers did not know how it would perform in patients with the disease. However, both an internal validation by the developer^10^ and a subsequent external validation^16^ on patients with COVID-19 revealed comparable if not superior DTI performance among those with COVID-19. For this study, we analyzed the DTI based on the manner in which it was likely to be implemented (ie, without accounting for COVID-19 status) to provide an assessment of its performance within the clinical practice setting.

In our bias assessment, we did not control for multiple protected features simultaneously, which limits our ability to understand how the DTI performs across specific intersectional groups (eg, patients who are young, of Black race, and female).^32^ Although we attempted such an analysis, the exponential increase in the number of subgroups when combining protected features led to many subgroups having no or few observations, making the results unreliable. New techniques to robustly assess intersectional bias are needed.^28^

## Conclusions

This prognostic study found that a proprietary deterioration model had poor to acceptable discrimination when applied to patient data from a large midwestern US health care system, and its performance generally decreased over longer lead times. Large tradeoffs were found between sensitivity and PPV as well as variable performance among certain demographic subgroups. These findings highlight the need for health care organizations to thoroughly evaluate the performance and equity of externally developed predictive models.

## Notes

Supplement 1. **eMethods.** The Epic Deterioration Index, Rationale for Observation-Level and Encounter-Level Analyses, and Confirmatory Records Review **eFigure 1.** Receiver Operating Characteristic and Precision Recall Curves **eFigure 2.** Calibration Plot of Observation-Level Predictions **eFigure 3.** Calibration Plot of Encounter-Level Predictions **eTable 1.** Ethnicities Included in *Other Ethnicity* Category **eTable 2.** Records Review Instrument
eReferences

Supplement 2.
Data Sharing Statement
