# Supplementary material for: Validation of a Proprietary Deterioration Index Model and Performance in Hospitalized Adults
Source: JAMA Netw Open. 2023 Jul 24;6(7):e2324176. doi: 10.1001/jamanetworkopen.2023.24176 (PMC10366696; doi:10.1001/jamanetworkopen.2023.24176)
Supplement: Supplement 1. — eMethods. The Epic Deterioration Index, Rationale for Observation-Level and Encounter-Level Analyses, and Confirmatory Records Review eFigure 1. Receiver Operating Characteristic and Precision Recall Curves eFigure 2. Calibration Plot of Observation-Level Predictions eFigure 3. Calibration Plot of Encounter-Level Predictions eTable 1. Ethnicities Included in Other Ethnicity Category eTable 2. Records Review Instrument eReferences [file jamanetwopen-e2324176-s001.pdf]

Supplementary Online Content

Byrd TF, Southwell B, Ravishankar A, et al. Validation of a proprietary deterioration index model and performance in hospitalized adults. *JAMA Netw Open*. 2023;6(7):e2324176. doi:10.1001/jamanetworkopen.2023.24176

eMethods. The Epic Deterioration Index, Rationale for Observation-Level and Encounter-Level Analyses, and Confirmatory Records Review

eFigure 1. Receiver Operating Characteristic and Precision Recall Curves

eFigure 2. Calibration Plot of Observation-Level Predictions

eFigure 3. Calibration Plot of Encounter-Level Predictions

eTable 1. Ethnicities Included in *Other Ethnicity* Category

eTable 2. Records Review Instrument

eReferences

This supplemental material has been provided by the authors to give readers additional information about their work.

© 2023 Byrd TF IV et al. *JAMA Network Open*.

eMethods. The Epic Deterioration Index, Rationale for Observation-Level and Encounter-Level Analyses, and Confirmatory Records Review

The Epic Deterioration Index

The model is an ordinal logistic regression model that categorizes patients into three risk buckets: green (low), yellow (medium), and red (high). The model developers decided to construct two linear combinations of clinical inputs that, using the proportional odds assumption, allows the feature space to be split into two decision boundaries. The target (a.k.a outcome or dependent variable) for model training was an ordinal integer: 2 if the patient dies in the next 38 hours (red risk), 1 if the patient does not die in the next 38 hours but is transferred to the ICU or has a rapid response team (RRT) or code called in the next 12 hours (yellow risk), and 0 if neither of the listed events occur (green risk). The model uses the following independent variables: age, systolic blood pressure, temperature, pulse, respiration rate, SpO2, several nursing assessments ('Glasgow Coma Score', 'abnormal neurologic assessment', 'abnormal cardiac rhythm', 'on oxygen'), hematocrit, WBC, potassium, sodium, blood pH, abnormal platelet count, and abnormal BUN.

Rationale for observation-level and encounter-level analyses

Observation- and encounter-level measurements are two distinct approaches to defining the performance of an early warning system, and each highlights different model strengths and weaknesses. At the observation level, each prediction (in this case made every fifteen minutes) counts equally. Therefore, a single high or low prediction is unlikely to change the overall performance. However, a string of high predictions wherein the outcome subsequently does not occur, as might be seen in a patient who has multiple advanced chronic diseases admitted for an extended time while awaiting placement, will bring down model performance. Besides giving excess weight to patients with longer hospitalizations, observation-level measurements do not take into account "muting" or ignoring subsequent predictions after an initial high-risk prediction.

In contrast, a single erroneous encounter-level prediction will bring down performance. By including only the highest deterioration score in encounter-level predictions, this analysis approximates the model's performance as if it was linked to an alert that only fires once during an admission - if the patient crossed a pre-defined high risk threshold. In the encounter-level analysis, each patient is represented by a single prediction, making it a more appropriate measurement for studying bias in smaller subgroups. This contrasts with the observation-level analysis, where a single patient could contribute hundreds of predictions, potentially skewing the analysis.

© 2023 Byrd TF IV et al. *JAMA Network Open*.

Confirmatory records review

We sampled encounters of patients in our study cohort who experienced any of the following events while hospitalized: mechanical ventilation, ICU transfer, mortality, vasopressor initiation, or comfort care initiation. Epic measures the performance of EDI retrospectively across hospitalizations by sampling predictions up to 38 hours before deterioration occurs. To match this time frame, we kept the highest EDI score made within 38 hours prior to the first event for each hospitalization. We binned these predictions into deciles so we could evenly sample the entire probability range of EDI scores. We then randomly selected five encounters from each score decile to create our final set of 50 hospitalizations for chart review.

Three physicians on the research team (BS, AR, TT) independently completed chart reviews and entered data into a REDCap electronic data capture tool hosted at the University of Minnesota.[1,2] We designed a custom data collection form to explore the timing of, events surrounding, and preventability of deterioration episodes, adapted from van Galen et al.[3] The form was comprised of a mix of discrete multiple-choice, date/time, Likert-style, and free-text items, as given in eTable 2. After independently reviewing the first ten cases, the team met and resolved areas of confusion and ambiguity through discussion. BS, AR, and TT then each reviewed all remaining cases (a total of three independent reviews per case). TB and AK audited select cases where the reviewers did not reach a consensus, made final determinations as appropriate, and compiled the data.

© 2023 Byrd TF IV et al. *JAMA Network Open*.

eFigure 1. Receiver Operating Characteristic and Precision Recall Curves

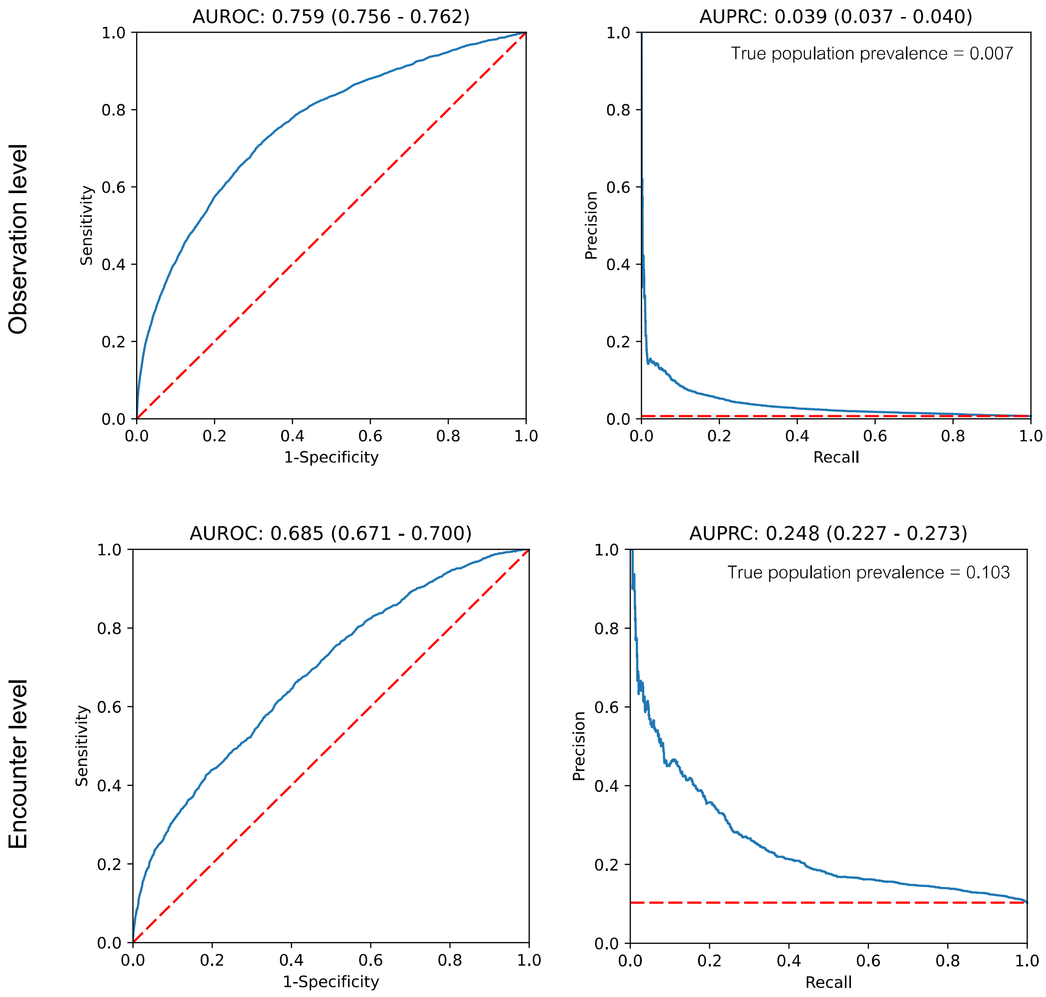

Overall DTI performance. AUROC = area under the receiver operation curve. AUPRC = area under the precision recall curve. Diagonal dashed lines: AUROC reference (AUROC of 0.5). Horizontal dashed lines: AUPRC reference (deterioration prevalence).

© 2023 Byrd TF IV et al. *JAMA Network Open*.

eFigure 2. Calibration Plot of Observation-Level Predictions

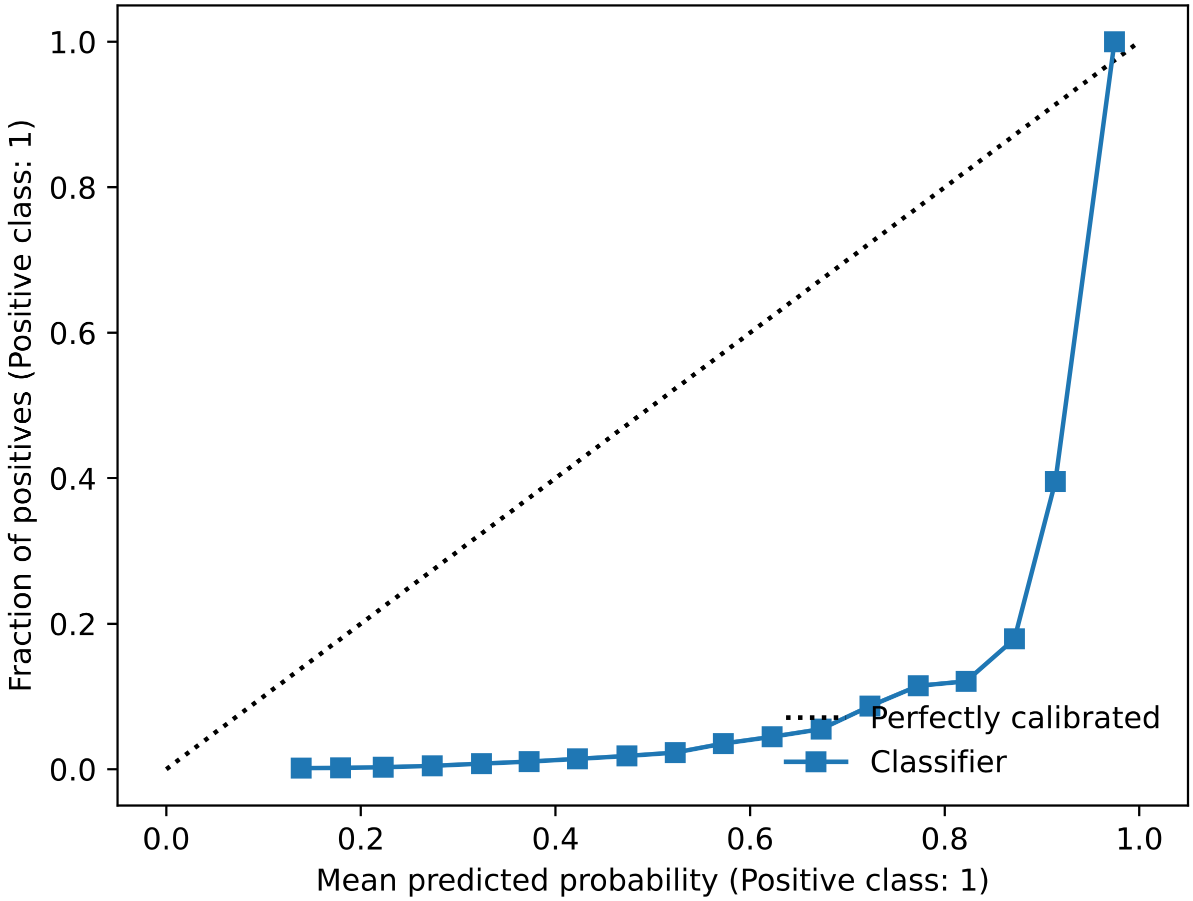

© 2023 Byrd TF IV et al. *JAMA Network Open*.

eFigure 3. Calibration Plot of Encounter-Level Predictions

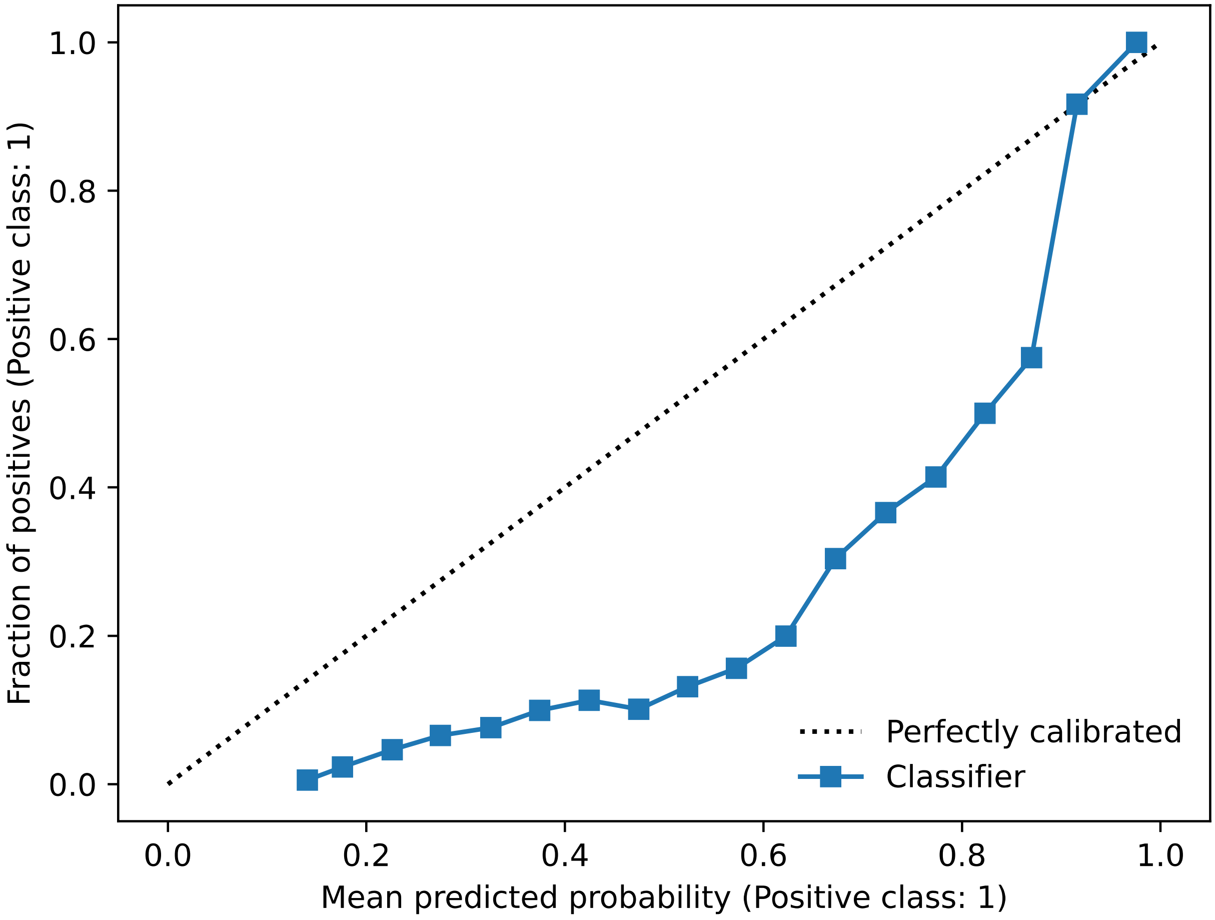

© 2023 Byrd TF IV et al. *JAMA Network Open*.

eTable 1. Ethnicities Included in *Other Ethnicity* Category

Ethnicity	n	%	Ethnicity	n	%
AFRICAN-AMERICAN	13	0.09	Israeli	1	0.01
African	20	0.14	Japanese	10	0.07
African American	11	0.08	Karen	2	0.01
American	5,424	38.97	Kenyan	21	0.15
Arabic	15	0.11	Korean	29	0.21
Asian	21	0.15	Laotian	20	0.14
Asian/Pacific Island	2	0.01	Latino	28	0.2
Bhutanese	2	0.01	Lebanese	9	0.06
Bosnia-Herzegovian	2	0.01	Liberian	16	0.11
Burmese	1	0.01	Mexican	89	0.64
Cambodian	17	0.12	Moroccan	3	0.02
Cameroon	6	0.04	Native Hawaiian/Other Pacific Island	2	0.01
Canadian	6	0.04	Nepalese	6	0.04
Caucasian	277	1.99	Nigerian	26	0.19
Chinese	46	0.33	Not Hispanic or Latino	4,664	33.51
Colombian	3	0.02	Oromoian	7	0.05
Congolese	2	0.01	Other	977	7.02
Croatian	8	0.06	Pakistani	4	0.03
Ecuadorian	10	0.07	Russian	39	0.28
Egyptian	7	0.05	Serbian	2	0.01
El Salvadorian	10	0.07	Sierra Leone	5	0.04
Eritrean	7	0.05	Somali	199	1.43
Ethiopian	61	0.44	South African	5	0.04
Filipino	20	0.14	Sudanese	8	0.06
Guatemalan	7	0.05	Thai	13	0.09
Hmong	51	0.37	Tibetan	4	0.03
Honduran	4	0.03	Ukranian	16	0.11
Indian	57	0.41	Vietnamese	56	0.4
Indonesian	1	0.01	native American/American Indian	13	0.09
Iranian	6	0.04			

© 2023 Byrd TF IV et al. *JAMA Network Open*.

eTable 2. Records Review Instrument

Data Dictionary Codebook

02/24/2023 12:42

	#	Variable / Field Name	Field Label<br>*Field Note*	Field Attributes (Field Type, Validation, Choices, Calculations, etc.)
Instrument: **Encounter Information** (encounter_information)				
	1	[mrn]	MRN	text, Identifier
	2	[hospital_id]	Hospital ID	text
	3	[department_name]	Department (at time of prediction)	text
	4	[time0]	Admission Datetime<br>*time0 is same as hosp_admsn_time_1 in acute datamart for these patients*	text (datetime_mdy)
	5	[hosp_disch_time_1]	Discharge Datetime	text (datetime_mdy)
	6	[score_dttm]	Prediction Datetime	text (datetime_mdy)
	7	[review_window_end]	38 hours after prediction	text (datetime_seconds_mdy)
	8	[encounter_information_complete]	Section Header: *Form Status*<br>Complete?	dropdown<br>0 Incomplete<br>1 Unverified<br>2 Complete
Instrument: **To Do: Deterioration Review** (to_do_deterioration_review)				
	9	[reviewer]	Who is performing this chart review?	dropdown, Required<br>1 Abhinab Kc<br>2 Adarsh Ravishankar<br>3 Bronwyn Southwell<br>4 Tom Byrd<br>5 Travis Tran
	10	[display_score_dttm]	Encounter details: MRN: [mrn] Admission: [time0] Discharge: [hosp_disch_time_1] PREDICTION WINDOW for chart review: [score_dttm] to [review_window_end]	descriptive
	11	[reason_for_admission]	Brief overview of admission (e.g., "admitted from nursing home w/ fall and hip fx found to have sepsis which was treated but required ICU transfer, eventually discharged home")	notes, Required
	12	[code_status]	Section Header: *Code Status*<br>Did patient have a code status of "DNR/DNI" any time during the prediction window?	yesno, Required<br>1 Yes<br>0 No
	13	[code_status_intubation]<br>Show the field ONLY if: [code_status] = '1'	Would the patient have been intubated during the prediction window if their code status had been "FULL CODE"?	radio, Required<br>1 Most likely<br>2 Unclear<br>3 Unlikely
	14	[code_status_comments]	Comments regarding patient's code status during the prediction window and its effect on clinicians' treatment of the patient's deterioration (e.g., would patient have been transferred to the ICU if they were not DNR/DNI status?)	notes
	15	[outcome_mortality]	Section Header: *Mortality*<br>Did patient die during the prediction window?	yesno, Required<br>1 Yes<br>0 No
	16	[mortality_comfort]<br>Show the field ONLY if: [outcome_mortality] = '1'	Was patient on comfort care and/or inpatient hospice at time of death?	yesno, Required<br>1 Yes<br>0 No

© 2023 Byrd TF IV et al. *JAMA Network Open*.

	17	[mortality_datetime]<br>Show the field ONLY if:<br>[outcome_mortality] = '1'	Time of death on death certificate	text (datetime_mdy), Required
	18	[mortality_location]<br>Show the field ONLY if:<br>[outcome_mortality] = '1'	Patient location at time of death (e.g. ED, medical floor, surgical floor, inpatient hospice, ICU)	text, Required
	19	[mortality_reason]<br>Show the field ONLY if:<br>[outcome_mortality] = '1'	Cause of death from death certificate	text, Required
	20	[mortality_prevent]<br>Show the field ONLY if:<br>[outcome_mortality] = '1' and [mortality_comfort] = '0'	Was death preventable?	radio, Required<br>1 Clearly preventable<br>2 Likely preventable<br>3 Unknown<br>4 Unlikely preventable<br>5 Clearly not preventable
	21	[mortality_comments]<br>Show the field ONLY if:<br>[outcome_mortality] = '1'	Comments on preventability of death	notes
	22	[outcome_icu]	Section Header: *ICU Transfer*<br>Was patient transferred to ICU during the prediction window?	radio, Required<br>1 Yes<br>2 No<br>3 Already in ICU
	23	[icu_from]<br>Show the field ONLY if:<br>[outcome_icu] = '1'	Where was patient transferred from (e.g. PACU, medicine service, ED)	text, Required
	24	[icu_to]<br>Show the field ONLY if:<br>[outcome_icu] = '1'	ICU patient was transferred to (e.g. MICU, SICU, etc.)	text, Required
	25	[icu_reason]<br>Show the field ONLY if:<br>[outcome_icu] = '1'	Reason for ICU transfer	notes, Required
	26	[icu_prevent]<br>Show the field ONLY if:<br>[outcome_icu] = '1'	Was ICU transfer preventable?	radio, Required<br>1 Clearly preventable<br>2 Likely preventable<br>3 Unknown<br>4 Unlikely preventable<br>5 Clearly not preventable
	27	[icu_comments]<br>Show the field ONLY if:<br>[outcome_icu] = '1'	Comments on preventability of ICU transfer	notes
	28	[outcome_vent]	Section Header: *Mechanical Ventilation*<br>Was patient intubated during the prediction window?	radio, Required<br>1 Yes<br>2 No<br>3 Already mechanically ventilated
	29	[vent_location]<br>Show the field ONLY if:<br>[outcome_vent] = '1'	Patient location at time of intubation (e.g. ED, medical floor, surgical floor, ICU)	text, Required
	30	[vent_reason]<br>Show the field ONLY if:<br>[outcome_vent] = '1'	Reason for intubation	notes, Required

© 2023 Byrd TF IV et al. *JAMA Network Open*.

	31	[vent_prevent]<br>Show the field ONLY if:<br>[outcome_vent] = '1'	Was intubation preventable?	radio, Required<br>1 Clearly preventable<br>2 Likely preventable<br>3 Unknown<br>4 Unlikely preventable<br>5 Clearly not preventable
	32	[vent_comments]<br>Show the field ONLY if:<br>[outcome_vent] = '1'	Comments on preventability of intubation	notes
	33	[outcome_arrest]	Section Header: *Cardiac Arrest*<br>Did patient have a cardiac arrest during the prediction window?	yesno, Required<br>1 Yes<br>0 No
	34	[arrest_datetime]<br>Show the field ONLY if:<br>[outcome_arrest] = '1'	Time of cardiac arrest (from scanned code blue document)	text (datetime_mdy), Required
	35	[arrest_location]<br>Show the field ONLY if:<br>[outcome_arrest] = '1'	Patient location at time of arrest (e.g. ED, medical floor, surgical floor, ICU)	text, Required
	36	[arrest_reason]<br>Show the field ONLY if:<br>[outcome_arrest] = '1'	Suspected cause of cardiac arrest	text, Required
	37	[arrest_prevent]<br>Show the field ONLY if:<br>[outcome_arrest] = '1'	Was cardiac arrest preventable?	radio, Required<br>1 Clearly preventable<br>2 Likely preventable<br>3 Unknown<br>4 Unlikely preventable<br>5 Clearly not preventable
	38	[arrest_comments]<br>Show the field ONLY if:<br>[outcome_arrest] = '1'	Comments on preventability of cardiac arrest	notes
	39	[outcome_rrt]	Section Header: *Rapid Response Team (RRT) Activation - RRT activation = bedside assessment of patient by primary or covering MD or APP, ICU nurse, and respiratory therapist. There may be a specific RRT note in the chart if RRT was activated.*<br>Was the rapid response team (RRT) activated during the prediction window?	yesno, Required<br>1 Yes<br>0 No
	40	[rrt_datetime]<br>Show the field ONLY if:<br>[outcome_rrt] = '1'	What time was RRT activated?	text (datetime_mdy)
	41	[rrt_comments]<br>Show the field ONLY if:<br>[outcome_rrt] = '1'	Comments on the effects of RRT activation on patient's trajectory	notes
	42	[covid_status]	Section Header: *COVID Status*<br>Was patient suspected or confirmed to be COVID positive during the prediction window?	yesno, Required<br>1 Yes<br>0 No
	43	[covid_cause]<br>Show the field ONLY if:<br>([outcome_mortality] = '1' or [outcome_icu] = '1' or [outcome_vent] = '1' or [outcome_arrest] = '1' or [outcome_rrt] = '1') and ([covid_status] = '1')	Was COVID the underlying cause of death, ICU transfer, cardiac arrest, mechanical ventilation, or rapid response team activation during the prediction window?<br>*if multiple of these occurred (e.g. ICU transfer then ventilated), only consider the first event (ICU transfer in this example)*	radio, Required<br>1 Yes<br>2 No<br>3 Unclear

	44	[outcome_sepsis]	Section Header: *Sepsis Presence*<br>Was bacterial sepsis newly suspected or treated during the prediction window? (Exclude sepsis exclusively from COVID i.e. no antibacterial treatment given)<br>*yes if "sepsis" or "suspected sepsis" listed as a problem in clinical note(s) and treatment plan included antibiotics*	radio, Required<br>1 Yes<br>2 No<br>3 Sepsis already suspected or being treated
	45	[sepsis_datetime]<br>Show the field ONLY if:<br>[outcome_sepsis] = '1'	Time of suspected sepsis or beginning of sepsis treatment<br>*could be first antibiotic administration or time of first note mentioning sepsis*	text (datetime_mdy), Required
	46	[sepsis_cause]<br>Show the field ONLY if:<br>([outcome_mortality] = '1' or [outcome_icu] = '1' or [outcome_vent] = '1' or [outcome_arrest] = '1' or [outcome_rrt] = '1') and ([outcome_sepsis] = '1' or [outcome_sepsis] = '3')	Was sepsis the underlying cause of death, ICU transfer, cardiac arrest, mechanical ventilation, or rapid response team activation during the prediction window?<br>*if multiple of these occurred (e.g. ICU transfer then ventilated), only consider the first event (ICU transfer in this example)*	radio, Required<br>1 Yes<br>2 No<br>3 Unclear
	47	[final_comments]	Section Header: *Additional Comments*<br>Any additional comments about this case	notes
	48	[to_do_deterioration_review_complete]	Section Header: *Form Status*<br>Complete?	dropdown<br>0 Incomplete<br>1 Unverified<br>2 Complete

© 2023 Byrd TF IV et al. *JAMA Network Open*.

eReferences

1. Harris PA, Taylor R, Minor BL, et al. The REDCap consortium: Building an international community of software platform partners. *Journal of Biomedical Informatics*. 2019;95:103208. doi:10.1016/j.jbi.2019.103208

2. Harris PA, Taylor R, Thielke R, Payne J, Gonzalez N, Conde JG. Research electronic data capture (REDCap)—A metadata-driven methodology and workflow process for providing translational research informatics support. *Journal of Biomedical Informatics*. 2009;42(2):377-381. doi:10.1016/j.jbi.2008.08.010

3. van Galen LS, Struik PW, Driesen BEJM, et al. Delayed Recognition of Deterioration of Patients in General Wards Is Mostly Caused by Human Related Monitoring Failures: A Root Cause Analysis of Unplanned ICU Admissions. *PLoS One*. 2016;11(8):e0161393. doi:10.1371/journal.pone.0161393

© 2023 Byrd TF IV et al. *JAMA Network Open*.
