# Supplementary material for: Validation of a Proprietary Deterioration Index Model and Performance in Hospitalized Adults
Source: JAMA Netw Open. 2023 Jul 24;6(7):e2324176. doi: 10.1001/jamanetworkopen.2023.24176 (PMC10366696; doi:10.1001/jamanetworkopen.2023.24176)
Supplement: Supplement 2. — Data Sharing Statement [file jamanetwopen-e2324176-s002.pdf]

Data Sharing Statement

Byrd, IV. Validation of a Proprietary Deterioration Index Model and Performance in Hospitalized Adults. *JAMA Netw Open*. Published July 24, 2023. doi:10.1001/jamanetworkopen.2023.24176

Data
Data available: No

Additional Information
Explanation for why data not available: Protected health information
